# SnO_2_ quantum dot decoration of CuO nanoparticles with enhanced NO_2_ and H_2_ gas sensing response *via* p–n heterojunction interfaces

**DOI:** 10.1039/d5ra05533d

**Published:** 2025-10-15

**Authors:** Valentina Paolucci, Thirugnanam Natarajan, Vittorio Ricci, Fabiola Ferrante, Carlo Cantalini

**Affiliations:** a Department of Industrial and Information Engineering and Economics, University of L'Aquila I-67100 L'Aquila Italy; b UdR INSTM of L'Aquila 67100 L'Aquila Italy

## Abstract

Design and fabrication of heterostructures has emerged as a powerful strategy to improve gas sensing performances compared to single materials counterparts. In this work, we report an innovative CuO-based nanostructure decorated with SnO_2_ quantum dots (QDs) for the detection of NO_2_ and H_2_ gases. Here, CuO serves as the base material while SnO_2_ QDs are used as the decorating phase: an inversion of the conventional architecture where SnO_2_ is typically the host and CuO the modifier. The composite exhibits higher sensitivity compared to pristine CuO and SnO_2_, showing state-of-the-art performances in terms of relative responses (RRs) in the 20 ppb to 1 ppm range and 10 ppm to 250 ppm for NO_2_ and H_2_ respectively, with excellent stability and reproducibility. Moreover, the SnO_2_-QDs/CuO operates at a low working temperature (*i.e.* 100 °C), offering significant advantages in terms of energy efficiency and material stability. The observed enhancements are attributed to the optimized heterointerface, increased active surface area, and modulation of the charge carrier induced by the p–n heterojunctions. These results highlight the potential of reverse-configured SnO_2_/CuO as a versatile platform for improved, low-temperature gas sensors with high sensitivity.

## Abstract 2

A novel SnO_2_-QDs/CuO nanocomposite, with CuO as base and SnO_2_ as modifier, is used for NO_2_ (20 ppb to 1 ppm) and H_2_ (10–250 ppm) sensing at 100 °C. The presence of p–n heterojunctions facilitates charge transfer and increases sensitivity.

## Graphical abstract

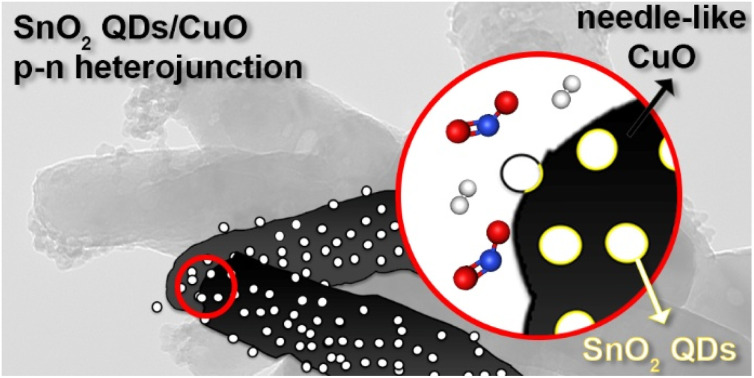

## 1. Introduction

The detection of toxic and explosive gases such as nitrogen oxide (NO_2_) and hydrogen (H_2_) is a critical requirement in the fields of health and environmental monitoring and industrial safety. Specifically, NO_2_ is an air pollutant primarily originating from combustion processes associated with vehicle traffic and industrial activities, which can cause serious respiratory diseases even at low concentrations.^1,2^ On the other hand, H_2_ gas, being colourless and odourless, poses a significant risk due to its wide flammability range (4–75% in air), low ignition energy (0.017 mJ) and high combustion heat (142 kJ per g H_2_), negatively influencing its exploitation as a promising fuel for the transition to a low carbon economy.^3,4^ To address these challenges, the development of sensitive and reliable gas sensors for detection of trace levels of NO_2_ and H_2_ remains an active area of research. Thin films of metal oxide semiconductors (MOX) have been widely employed as chemo-resistive gas sensors due to their high sensitivity, low cost and simple fabrication processes. Typically, n-type MOX materials such as SnO_2_, ZnO and In_2_O_3_,^5–7^ and p-type CuO, NiO and Cr_2_O_3_ counterparts^8–10^ have been widely studied for gas sensing applications. However, one major limitation is their high operating temperature, often exceeding 200 °C, which increases power consumption and hinders their integration in portable or wearable devices.^11–13^

Recent studies have demonstrated that carefully engineered oxide heterostructures can significantly enhance charge separation and gas adsorption processes.^14,15^ In particular, a promising strategy is represented by the design of heterojunctions combining p-type and n-type metal oxides, leveraging the formation of p–n junctions to improve gas sensing at lower temperatures.^16,17^ Among p-type materials, CuO stands out due to its excellent surface reactivity, stability and ability to form hierarchical nanostructures in the form of flakes, nanorods and nanofibers.^8,18–20^ On the other hand, SnO_2_, being one of the first MOX sensor ever studied,^21^ is well known for its strong interactions with both oxidizing and reducing gases.^22,23^ The combination of CuO and SnO_2_ has been explored extensively, typically in the configuration where SnO_2_ serves as the base material and CuO is introduced as the secondary phase.^24–27^ These structures, showing typical n-type response, have been extensively utilized for H_2_S sensing on account of the reversible conversion of CuO in CuS, which significantly reduces the resistance.^28^ In this work, we propose a reverse composite configuration, employing CuO as the primary sensing matrix and decorating it with SnO_2_ quantum dots (QDs). This architecture leverages the predominant p-type response of CuO-based interfaces to oxidizing gases like NO_2_,^29^ while promoting intimate contact between the SnO_2_ QDs edges and the underlying CuO surface, resulting in enhanced interfacial charge transfer and gas sensing performance.^30^ Remarkably, this SnO_2_-QDs/CuO architecture exhibits superior sensing performance towards both NO_2_ and H_2_ at a reduced operating temperature of 100 °C. Specifically, needle-like CuO was synthesized *via* microwave irradiation of Cu_2_(OH)_2_CO_3_, while SnO_2_ QDs were produced using a colloidal solution method. The formation of nanoscale p–n junctions at the SnO_2_/CuO interfaces, enhancing charge carrier separation and modulation of the depletion layer, is responsible to improve gas sensing response.^17^ Furthermore, the choice of SnO_2_ in quantum dots form introduces significant advantages. Due to its nanoscale size and high surface-to-volume ratio, SnO_2_ provides more active sites for gas adsorption, easing faster charge transfer kinetics.^31,32^ Moreover, the quantum confinement effect also modulates the electronic properties of the heterojunction, enhancing sensitivity at low temperatures (*i.e.* 100 °C). Overall, this study provides a novel perspective on CuO-based heterojunctions, proposing a simple and scalable synthesis route that make this approach suitable for practical application, demonstrating enhanced NO_2_ and H_2_ sensing performances at low temperatures, thus offering a promising alternative to conventional single-phase or inversely configured systems.

## 2. Experimental

### 2.1 Synthesis of CuO nanostructures

The hydrothermal synthesis of Cu_2_(OH)_2_CO_3_ was performed according to a reported procedure:^33^ 4.2 ml of 0.4 M copper(ii) acetate and 4.2 ml of 0.8 M urea were dissolved into 3.3 ml DI water. After 30 min stirring, the mixture was transferred into a 40 ml Teflon-lined stainless-steel autoclave and maintained at 120 °C for 4 h. After that, the autoclave was naturally cooled down to room temperature and the obtained product was washed 5 times with DI water and 3 times with absolute ethanol and dried at 50 °C for 1 h. Then 0.1 g of Cu_2_(OH)_2_CO_3_ were added to 20 ml of DI water, and the obtained suspension was irradiated with microwave at 560 W for 5 minutes. After collecting and washing, a brown precipitate was obtained. Finally, the powder was annealed in a muffle furnace at 400 °C for 2 h with a heating rate of 1 °C min^−1^ to obtain crystalline CuO. A detailed flowchart of the process is reported in SI S1a.

### 2.2 Synthesis of SnO_2_ QDs

50 ml 0.025 M ammonia solution 30% was added dropwise into 50 ml 0.05 M SnCl_4_·5H_2_O solution and kept stirring at 80 °C in oil bath for 3 h.^34^ At the end of the reaction, the resulting white gel-like product was collected by centrifugation at 5000 rpm and washed five times with DI water to remove any residual precursors. The precipitate was then dried overnight at 60 °C. For further characterization, the dried SnO_2_ product was redispersed in ethanol for deposition and analysis. A detailed flowchart of the process is reported in SI S1b.

### 2.3 Decorating CuO nanoparticles with SnO_2_-QDs

The optimized procedure for SnO_2_ decoration of CuO flakes was established through systematic variation of precursor concentration and order of addition (see SI, Fig. S2). In the final protocol, 25 mg of synthesized crystalline CuO were added to 20 ml of DI water; then, 2.6 mg SnCl_4_·5H_2_O powder and 0.3 ml of 0.05 M ammonia solution were successively incorporated to the dispersion. After mixing, obtained dispersion was heated at 80 °C using an oil bath and maintained under stirring for 3 h. The obtained product was washed three times by centrifugation at 5000 rpm, followed by a final centrifugation at the same speed to exchange the solvent with ethanol, facilitating solvent evaporation after deposition and enhancing both microstructural and electrical characterization. A detailed flowchart of the process is reported in SI S1c.

Notably, the hydrothermal synthesis of Cu_2_(OH)_2_CO_3_ typically afforded a yield of ∼43% (∼130 mg of Cu_2_(OH)_2_CO_3_ from ∼300 mg of copper(ii) acetate). In contrast, both the SnO_2_ and the SnO_2_/CuO syntheses showed essentially quantitative conversion of the Sn precursor, as no secondary phases or unreacted material were detected by our structural analyses.

### 2.4 Material characterization

The crystal structure was analysed through the X-ray diffraction Grazing Incidence X-ray Diffraction (GI-XRD) by XRD-PANAnalytical X’PERT Pro using Cu Kα_1_ radiation (*λ* = 1.5406 Å) with an incident angle of 0.8°. FTIR spectra were obtained by Thermo Nicolet Nexus 870, operating in the 400–4000 cm^−1^ spectral range. Thermogravimetric and differential thermal analysis (TG-DTA) was performed in air atmosphere using a Linseis L81-I. The morphology was studied by Transmission Electron Microscopy Philips CM100 operating at 100 kV and Scanning Electron Microscopy Gemini SEM working at 5 kV. HRTEM was performed using a Talos F200S. The optical absorption and reflectance spectra were measured using a PerkinElmer LAMBDA 1050+ UV-vis-near-infrared (NIR) spectrophotometer and in diffuse reflectance spectroscopy (DRS) configuration respectively, and the Kubelka–Munk function was utilized.

### 2.5 Electrical characterization

CuO, SnO_2_ and SnO_2_-QDs/CuO based films were deposited by spin coating on Si/Si_3_N_4_ substrates, with comb-like Pt interdigitated electrodes (30 μm apart) and back side heaters and inserted in a 500 cm^3^ Teflon chamber for chemoresistive gas sensing characterization. By stepwise tuning the current in the back side heater, sensor's substrates are almost instantaneously kept and maintained at the selected operating temperature. Electrical responses were collected using an automated volt–amperometric system (Agilent 34970A), measuring electrical resistance of the film at operating temperatures from 25 to 150 °C and different environments. Gas concentrations were obtained by diluting NO_2_ (10 ppm in air) and H_2_ (500 ppm in air) certified mixtures (Nippon gases-IT) with synthetic dry air utilizing mass flow controllers (MKS147), setting the total flow rate at 500 sccm per min. 50% relative humidity (RH%) was obtained by mixing dry with water-saturated air at 25 °C and measuring RH% at 25 °C before injection into the test chamber (Thermohygrometer – Hannah Instruments).

To characterize and compare gas responses properties of the different samples, the following definitions apply: (i) baseline resistance (BLR): the resistance in dry air at equilibrium; (ii) relative response (RR): defined as (*R*_Air_/*R*_Gas_) or (*R*_Gas_/*R*_Air_) depending on the oxidizing/reducing nature of the gases; (iii) sensor's sensitivity (*S*): the slope of the calibration curve (*i.e.* RR *vs.* gas concentration); (iv) response time (*τ*_ADS_) and recovery time (*τ*_DES_) defined as the time required for the resistance to reach 90% of the equilibrium value after injecting the gas and the time needed to return to 10% above the original value in air, respectively.

## 3. Results and discussion

We have applied three different chemical syntheses to prepare: (i) needle-like CuO nanoparticles; (ii) SnO_2_-QDs; and (iii) SnO_2_-QDs decorated needle-like CuO (SnO_2_-QDs/CuO). The detailed flowcharts of processes (i)–(iii) are reported in SI S1. Notably, while the CuO and SnO_2_ syntheses followed established literature routes,^33,34^ the synthesis of SnO_2_-QDs/CuO was the result of an optimization process involving both the relative amount of precursors and the order of their addition, as extensively described in SI S2. All material interfaces, deposited on dedicated Si_3_N_4_ substrates provided with Pt finger-type electrodes and a backside heater, have been investigated as NO_2_ and H_2_ sensors under dry and humid air background conditions.

### 3.1 Synthesis and microstructural characterization

#### 3.1.1 Needle-like CuO nanoparticles

**Fig. 1:**
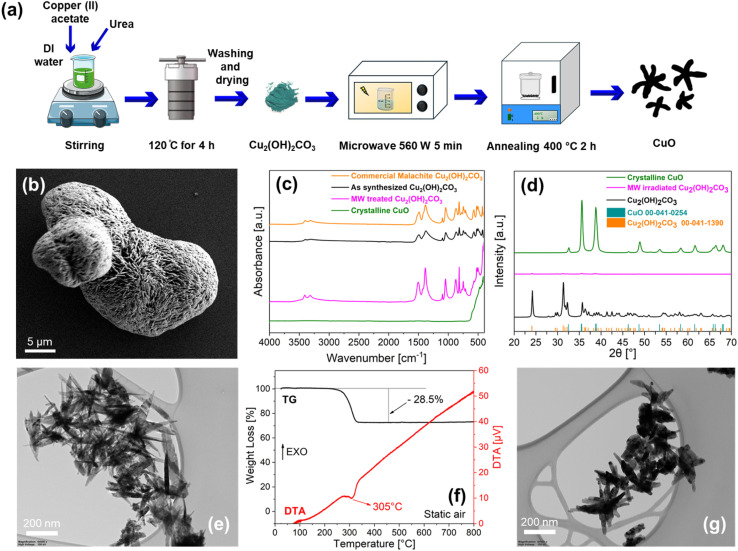
(a) Schematics of the synthesis process: after hydrothermal synthesis of Cu_2_(OH)_2_CO_3_ at 120 °C for 4 h, powder was dispersed in DI water and irradiated with microwave (MW) at 560 W for 5 min and annealed at 400 °C for 2 h to obtain crystalline needle-like CuO; (b) SEM image of Cu_2_(OH)_2_CO_3_ after hydrothermal synthesis; (c) comparison of the FT-IR spectra of: commercial malachite (Cu_2_(OH)_2_CO_3_, orange line), Cu_2_(OH)_2_CO_3_ after hydrothermal synthesis (black line), microwave irradiated (pink line) and annealed (green line) Cu_2_(OH)_2_CO_3_; (d) XRD spectra of Cu_2_(OH)_2_CO_3_, microwave irradiated and annealed Cu_2_(OH)_2_CO_3_ with associated JCPDS cards; (e) TEM image of needle-like Cu_2_(OH)_2_CO_3_ after MW; (f) thermogravimetric (TG) and differential thermal analysis (DTA) plots of MW Cu_2_(OH)_2_CO_3_ powder heated in air at 5 °C min^−1^ from 25 to 800 °C. Black and red lines refer to TG and DTA signals, respectively; (g) TEM image of needle-like crystalline CuO after annealing.

Following the procedure described in the Experimental section and Fig. 1a and SI S1a, the hydrothermal synthesis utilizing copper(ii) acetate and urea solution leads to the formation of round-shaped agglomerated-particles with chemical composition Cu_2_(OH)_2_CO_3_. Specifically, Scanning Electron Microscopy (SEM) (Fig. 1b), exhibits the formation of hierarchical microspheres made of “flower-like” assembled-nanosheets, growing radially from the core of the particle, with similar features as those described in literature.^33^ According to Fig. 1c, the chemical composition of the synthesized “flower-like” structures (black line), correspond to Cu_2_(OH)_2_CO_3_ as observed by comparing the Fourier Transform Infrared (FT-IR) spectrum of the synthesized structures with that of a commercial malachite powder (orange line). X-ray Diffraction (XRD) analysis (Fig. 1d) confirms that Cu_2_(OH)_2_CO_3_ flower-like particles are highly crystalline (black line of Fig. 1d), consistent with JCPDS Card No. 00-041-1390 corresponding to malachite.

The “flower-like” Cu_2_(OH)_2_CO_3_ agglomerated structure (Fig. 1a), after microwave (MW) treatment at 560 W for 5 minutes, separates into “needle-like” free nanoparticles as those displayed in Fig. 1e, while maintaining the same chemical composition of Cu_2_(OH)_2_CO_3_ (pink line of Fig. 1c). Surprisingly, the XRD pattern of the microwave synthesized needle-like Cu_2_(OH)_2_CO_3_ particles, reveals the absence of distinct diffraction peaks (pink line of Fig. 1d), suggesting that the microwave treatment induces amorphization of the particles, while preserving their original Cu_2_(OH)_2_CO_3_ chemical composition. Finally, the amorphous “needle-like” Cu_2_(OH)_2_CO_3_ particles were subjected to annealing in dry air, to promote the formation of crystalline CuO. Preliminary simultaneous thermogravimetric (TG) and differential thermal analysis (DTA) technique was utilized to determine the most favourable annealing temperature.

Specifically, heating the amorphous “needle-like” Cu_2_(OH)_2_CO_3_ at 5 °C min^−1^ from 25 to 400 °C in a simultaneous TG-DTA apparatus, the thermogravimetric (TG) curve (black curve of Fig. 1f) yields a weight loss of −28.5% with a maximum decomposition rate at 305 °C (red line of the DTA signal of Fig. 1f). Remarkably, the measured weight loss of −28.5%, satisfactorily matches, within the experimental error, the theoretical weight loss of −30.5%, corresponding to the complete conversion of Cu_2_(OH)_2_CO_3_ into CuO, based on the following reaction:Cu_2_(OH)_2_CO_3(s)_ → 2CuO_(s)_ + CO_2(g)_ + H_2_O_(g)_

Consequently, after oven annealing at 400 °C for 2 h in static air, the “needle-like” amorphous Cu_2_(OH)_2_CO_3_ particles are isomorphically converted into “needle-like” CuO nanoparticles (Fig. 1g). Moreover, XRD characterization (green line of Fig. 1d) exhibits that CuO nanoparticles are highly crystalline (JCPDS Card 00-041-0254) and almost pure, as confirmed by FTIR analysis (green line of Fig. 1c). In conclusion, congruent with previous research,^33,35^ we synthesized pure and highly crystalline needle-like CuO nanoparticles to be utilized as scaffolds for SnO_2_-QDs decoration.

#### 3.1.2 SnO_2_-QD synthesis and SnO_2_-QD/CuO nanoparticles decoration

**Fig. 2:**
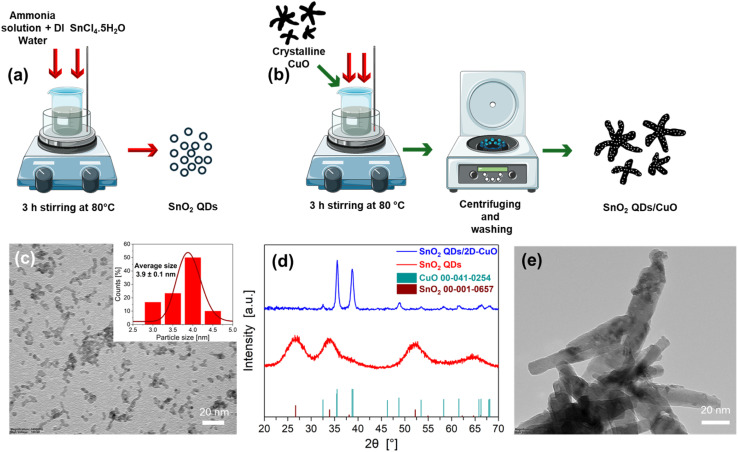
(a) Synthesis of SnO_2_ QDs and (b) SnO_2_-QDs/CuO; (c) TEM image of SnO_2_ QDs with inset reporting particle size distribution; (d) XRD spectra of SnO_2_ QDs and SnO_2_-QDs/CuO with associated JCPDS cards; (e) TEM image of SnO_2_-QDs/CuO.

The SnO_2_ Quantum Dots (SnO_2_-QD) synthesis,^34^ shown in Fig. 2a and described in Experimental section and SI S1b, yields agglomerated spherical SnO_2_ nanoparticles (TEM Fig. 2c) with average radii of *R* = 1.9 ± 0.1 nm and diameter-size distribution shown in the inset of Fig. 2c. XRD analysis of the synthesized SnO_2_-QDs nanoparticles, exhibits the formation of low-crystalline SnO_2_ structures, as demonstrated by the presence of broad diffraction peaks in the red curve of Fig. 2d, matching rutile phase of tetragonal tin oxide (JCPDS Card No. 00-001-0657). According to this procedure, we successfully synthesized SnO_2_ nanoparticles, with average radii (*R* = 1.9 ± 0.1 nm) smaller than Bohr's exciton radius (2.7 nm) in SnO_2_-QDs,^34,36^ attesting the capability of the synthesized SnO_2_-QDs to yield quantum confinements effects.

**Fig. 3:**
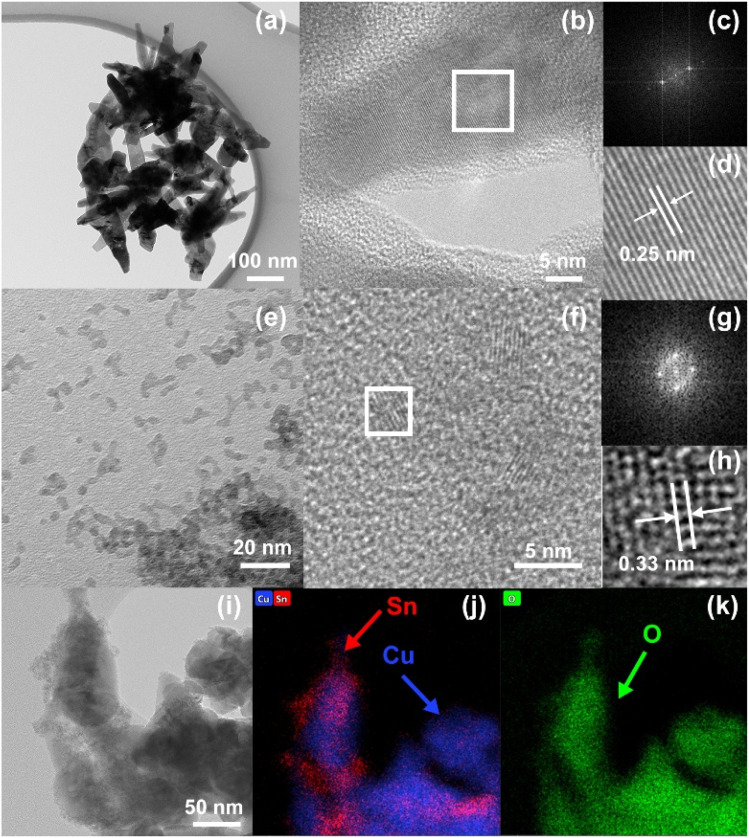
(a)–(d) TEM characterization of not decorated needle-like CuO: (a) low resolution TEM showing CuO microstructure, (b)–(d) high resolution TEM showing well-ordered crystalline structure (e)–(h) TEM characterization of as synthesized SnO_2_ QDs: (e) low resolution TEM showing dots microstructure, (f)–(h) high resolution TEM showing the small crystalline domains of SnO_2_ QDs; (i) high resolution TEM of SnO_2_-QDs/CuO and EDX maps (j) and (k) with indicated the elemental composition.

The SnO_2_-QDs decoration of the CuO needle-like nanoparticles to yield SnO_2_-QD/CuO, was carried out according to an optimized procedure (Fig. 2b and SI S1, S2) by mixing previously prepared needle-like CuO in 20 ml DI water with 2.6 mg SnCl_4_·5H_2_O and 0.3 ml of 0.05 M ammonia solution (Fig. 2b) to yield, after centrifugation and washing, decorated SnO_2_-QDs/CuO. TEM analysis of the SnO_2_-QDs/CuO, shown in Fig. 2e, exhibits the formation of small spherical SnO_2_ QDs nanoparticles of ≈4 nm size over the edge of the CuO needle-like structures. The XRD pattern of the SnO_2_-QD/CuO (blue line of Fig. 2d) confirms the presence of crystalline CuO phase, but it does not display any discernible reflections belonging to SnO_2_-QDs, possibly on account of a limited quantity of the SnO_2_-QDs phase as respect to the CuO matrix. HRTEM characterizations of the CuO needle-like, SnO_2_-QDs and SnO_2_-QDs/CuO, shown in Fig. 3, highlight that CuO needle-like nanoparticles are highly crystalline (Fig. 3a–d), comprising well-ordered lattice fringes with interplanar spacing of 0.25 nm (Fig. 3d), corresponding to the (002) plane of monoclinic CuO.^33^

In a similar way, HRTEM of SnO_2_ QDs (Fig. 3e–h), display the occurrence of crystalline domains (*i.e.* inside the white box of Fig. 3f), with average diameter's size smaller than 5 nm and interplanar spacing of 0.33 nm (Fig. 3h), consistent with the (110) plane of tetragonal SnO_2_. The effectiveness of the SnO_2_ QDs decoration of CuO needle-like nanoparticles is finally confirmed by HRTEM of Fig. 3i and by the associated EDX elemental map of the SnO_2_-QDs/CuO composite (Fig. 3j and k), attesting a congruent distribution of Cu (cyan), Sn (red) and O (green) elements over the CuO needle-like scaffold.

**Fig. 4:**
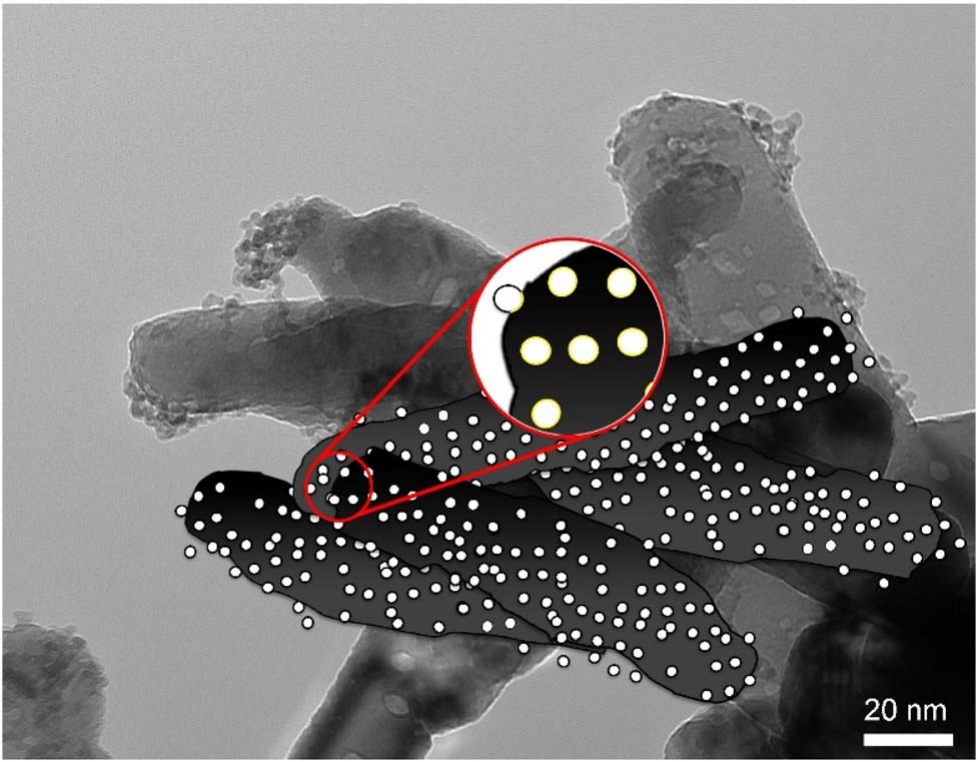
TEM image with superimposed a schematic illustration of the SnO_2_-QDs/CuO model structure. The SnO_2_ quantum dots (white) are evenly dispersed as isolated spots on the CuO surface (black). The overall structure consists of a network-like arrangement of needle-shaped CuO nanoparticles, which are decorated with SnO_2_ quantum dots.

Finally in Fig. 4 we have reported a schematization of the SnO_2_-QDs/CuO model structure. Congruently with HRTEM characterization (see Fig. 3), SnO_2_-QDs (white) are discretely distributed as isolated spots over the CuO underlying surface (black). The whole structure comprises a network morphology of needle like CuO nanoparticles decorated with SnO_2_-QDs.

### 3.2 Optical properties characterization

**Fig. 5:**
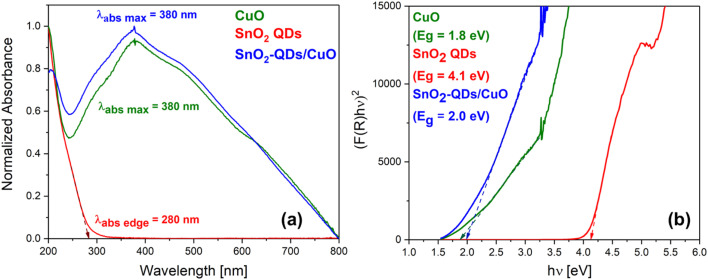
Optical characterization of CuO, SnO_2_ QDs and SnO_2_-QDs/CuO. (a) UV-vis absorption spectra (0.5 mg ml^−1^ in ethanol) and (b) Tauc plot with highlighted the measured bandgaps determination, respectively.

The optical absorbance of CuO needle-like nanoparticles, SnO_2_-QDs and SnO_2_-QDs/CuO was measured by UV-vis-near-infrared spectrophotometer as shown in Fig. 5a. Associated bandgaps, reported in Fig. 5b, were determined by DRS applying the Kubelka–Munk method.^37^ The CuO needle-like nanoparticles absorption curve (green line of Fig. 5a), exhibits a maximum located at ≈380 nm, attributed to electrons band-gap excitation in CuO.^38^ SnO_2_-QDs (red line), on the other hand, highlight an absorption edge at ≈280 nm, blue shifted compared to that of bulk SnO_2_ (345 nm).^39^ According to literature,^40^ the blue shift may represent the quantum confinement effect associated to the synthesized SnO_2_-QDs. Finally, the absorption of SnO_2_-QDs/CuO represented by the blue line in Fig. 5a, does not show any change in the wavelength position compared to that of CuO, but its absorbance is increased considerably. This behavior can be associated to the change in CuO band gap due to SnO_2_ decoration and to the formation of surface defects.^25^

The bandgaps of CuO needle-like nanoparticles, SnO_2_-QDs and SnO_2_-QDs/CuO displayed in Fig. 5b were extrapolated by Tauc’s plot^41^ using Kubelka–Munk^37^ function from diffuse reflectance spectra (E1):

E1 (*F*(*R*)*hv*)^1/*γ*^ = *B*(*hv* − *E*_g_)

here *h* [kg m^2^ s^−1^] is Planck's constant, *ν* [s^−1^] is the frequency of the incident electromagnetic radiation, *E*_g_ [eV] is the optical bandgap energy, *B* is a constant and *γ* = 1/2, corresponding to a direct band transition for CuO, SnO_2_-QDs and SnO_2_-QDs/CuO. The measured bandgap (BG) of 1.8 eV found for CuO needle-like nanoparticles (green line of Fig. 5b), is different from that of bulk CuO (1.24 eV) and closer to the reported BG value of 1.73 eV of CuO nanopetals.^42^ The 4.1 eV measured bandgap of SnO_2_-QDs (red line of Fig. 5b), which is consistently higher than that of bulk SnO_2_ (3.6 eV),^34^ can be possibly explained considering the QDs nature of the synthesized SnO_2_-QDs nanoparticles as respect to bulk SnO_2_. To better clarify the nature of this mismatch, we estimated the SnO_2_-QDs bandgap using Brus's variational method,^43^ according to eqn (E2),E2
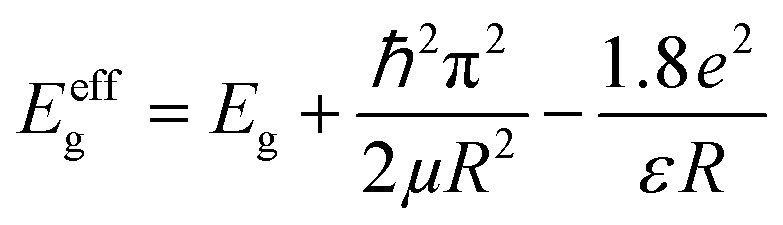
where *E*_g_ is the bulk band gap energy (3.6 eV), *ℏ* is reduced Planck's constant, *μ* is the effective reduced mass expressed as 
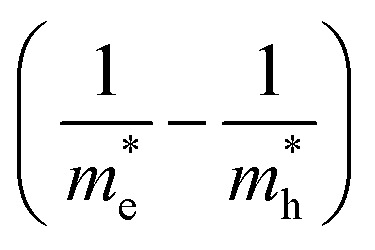
 and *μ* may be replaced by the electron effective mass (
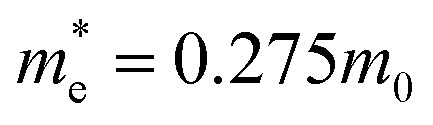
), since 
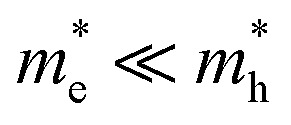
 (
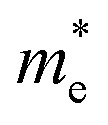
 and 
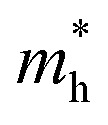
 are the electron and hole effective masses, respectively).^44^*R* is the radius of QDs, *e* is a charge of an electron (1.602 × 10^−19^ C) and *ε* is the dielectric constant (for SnO_2_, *ε* = 14). Hence, the calculated band gap energy using eqn (E2) is ∼4.0 eV, which is close to the measured band gap of 4.1 eV (Fig. 5b), confirming the quantum confinement effect occurring in the synthesized SnO_2_ QDs.^34^ Finally, the bandgap of SnO_2_-QDs/CuO is determined as 2.0 eV, which is higher than that of CuO needle-like nanoparticles (*E*_g_ = 1.8 eV), with this increment again related to the presence of SnO_2_-QDs,^24^ as previously discussed.

### 3.3 Gas sensing characterization

**Fig. 6:**
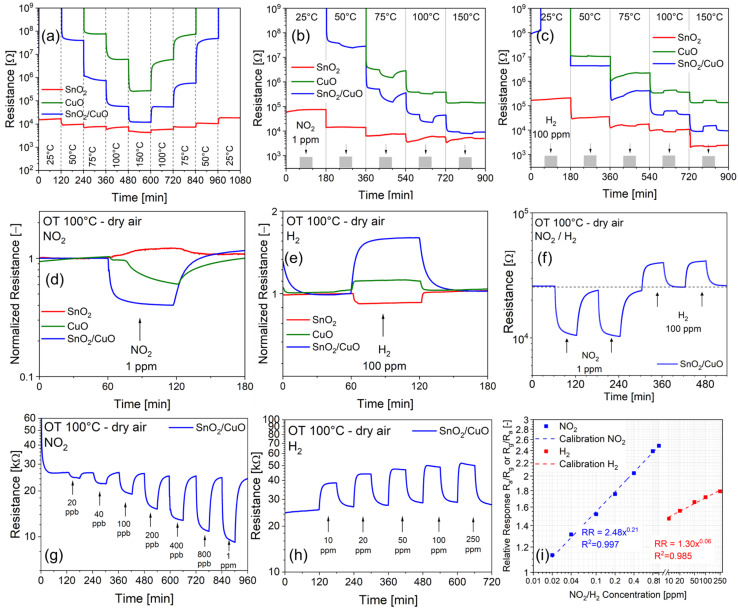
(a) BLR modulation of CuO (green), SnO_2_ (red) and SnO_2_-QDs/CuO (blue) measured in dry air under increasing/decreasing the OT in the 25–150–25 °C range; (b) gas sensing response to 1 ppm NO_2_ and (c) to 100 ppm H_2_ at different OTs (25–150 °C); (d) and (e) comparison of the normalized gas response to 1 ppm NO_2_ and 100 ppm H_2_ at 100 °C OT; (f) SnO_2_-QDs/CuO reproducibility response to NO_2_ (1 ppm) and H_2_ (100 ppm) at 100 °C OT; (g) and (h) dynamic electrical responses of the SnO_2_-QDs/CuO in dry air at an OT of 100 °C to NO_2_ (20 ppb to 1 ppm) and H_2_ (10 ppm to 250 ppm); (i) SnO_2_-QDs/CuO log/log calibration plots at 100 °C OT of the sensor's signal (*i.e.*, RR = *R*_a_/*R*_g_ or *R*_g_/*R*_a_) *vs.* NO_2_ and H_2_ gas concentrations.

The semiconducting nature of the CuO needle-like nanoparticles, SnO_2_-QDs and SnO_2_-QDs/CuO heterojunction is attested by the decrease/increase of the baseline resistance (*i.e.* BLR – the resistance at equilibrium in dry air), when increasing/decreasing the operating temperature (OT) between 25 °C and 150 °C, as shown in Fig. 6a. CuO nanoparticles and SnO_2_-QDs/CuO (green and blue lines) show higher baseline resistances compared to pristine SnO_2_-QDs (red line). The BLR of CuO nanoparticles is displayed departing from 75 °C, since at *T* < 75 °C the material's resistance exceeds the instrumental capabilities. Oppositely, the base line resistance (BLR) of SnO_2_-QDs is smaller than the others with BLR's modulation limited in the range 4–20 kΩ. The lower BLR of the SnO_2_-QDs film can be related to a size quantization effect, which has recently been demonstrated to be responsible for the increase in conductivity, when the size of the SnO_2_ nanoparticles is lowered within the nanometer scale.^45^ The SnO_2_-QDs/CuO shows BLR values that are in between those of CuO nanoparticles and SnO_2_-QDs, demonstrating the effectiveness of the SnO_2_ decoration to tune the film conductivity.

To evaluate the gas sensing properties, concentration ranges of 0.020–1 ppm for NO_2_ and 10–250 ppm for H_2_, were selected. This choice reflects the significantly higher electron affinity of NO_2_ (2.3 eV) compared to H_2_ (0.18 eV),^46,47^ which leads to a more pronounced sensor signal variation for NO_2_ at equivalent concentrations.

To evaluate the optimal operating temperature (OT) of each sensor, films were exposed to low concentrations of NO_2_ (1 ppm) and H_2_ (100 ppm) in dry air by varying the temperature in the range of 25–150 °C, as shown in Fig. 6b and c. At 25 °C OT, the base line resistance moves beyond 10^9^ Ω exceeding the instrumental capabilities. By stepwise increasing the operating temperature from 25 °C to 150 °C (Fig. 6b and c) the base line resistance of all sensors decreases, consistent with their semiconducting nature (see also Fig. 6a). Specifically, as shown in Fig. 6b, starting from 75 °C the introduction of 1 ppm NO_2_ into the test chamber (indicated by the grey rectangle at the bottom), causes a noticeable decrease of the resistance for both the CuO (green) and SnO_2_-QDs/CuO (blue) sensors. Opposedly, base line resistance of SnO_2_ remains largely unaffected upon exposure to NO_2_. Conversely, as indicated in Fig. 6c, when 100 ppm H_2_ is introduced into the chamber, the resistance of both CuO (green) and SnO_2_-QDs/CuO (blue) increases. It turns out that both CuO nanoparticles and SnO_2_-QDs/CuO exhibit p-type behavior, associated to a decrease/increase of the resistance upon NO_2_/H_2_ exposure, congruently with previous studies on CuO^48^ and CuO-based heterostructure sensors.^49,50^ Opposedly, SnO_2_ quantum dots (QDs) demonstrate typical n-type response, characteristic of SnO_2_ metal oxide sensors,^51^ with a negligible resistance increase/decrease to NO_2_/H_2_.

Considering now that the onset gas sensing temperature to NO_2_/H_2_ of the SnO_2_-QDs/CuO heterojunction is 50/100 °C, while that of SnO_2_-QD and CuO is 100 °C (Fig. 6b and c), we set the operating temperature (OT) at 100 °C, as a balance between the sensor's signal amplitude (here referred as the relative response, RR) and a fast and reversible baseline recovery. Specifically, sensors' electrical responses at 100 °C to 1 ppm NO_2_ and 100 ppm H_2_ in dry air are shown in Fig. 6d and e. We found that at 1 ppm NO_2_, the RRs values (*R*_a_/*R*_g_ or *R*_g_/*R*_a_, depending on the p/n nature of the sensor) are 1.6, 2.5, and 1.2 for CuO, SnO_2_-QDs/CuO and SnO_2_-QDs (with an estimated standard deviation of ±0.1 calculated over a set of 5 identical measurements). RRs to 100 ppm H_2_ are slightly smaller: 1.2, 1.7, and 1.1, confirming the superior performances of the SnO_2_-QDs/CuO to detect both NO_2_ and H_2_. As a concluding remark, the SnO_2_-QD/CuO shows excellent reverse capability to measure NO_2_ and H_2_ with fast and reversible base line recovery, as displayed in Fig. 6f.

Fig. 6g and h show the dynamic resistance changes of the SnO_2_-QDs/CuO to NO_2_ in the range 20 ppb to 1 ppm and H_2_ (10–250 ppm) in dry air background at 100 °C OT. SnO_2_-QDs/CuO shows a remarkable modulation of the sensor's signal to increasing NO_2_ and H_2_ gas concentrations, with excellent recovery of the BLR following each step of gas/dry air purge. The log–log calibration plots of the sensor's signal (*i.e.*, RR = *R*_a_/*R*_g_ or *R*_g_/*R*_a_) *vs.* NO_2_ and H_2_ gas concentrations shown in Fig. 6i, yield gas sensitivities (*S*), as represented by the slope of the calibration lines, *S*_NO_2__ = (0.21 ± 0.01) [ppm]^−1^ and *S*_H_2__ = (0.06 ± 0.01) [ppm]^−1^. The limits of detections (LOD) for NO_2_ and H_2_ were determined by extrapolating the calibration lines in Fig. 6i to the point where the response ratio (RR) equals 1, yielding LOD_(NO_2_)_ = 12 ppb and LOD_(H_2_)_ = 15 ppb respectively. The better NO_2_ dynamic responses (RRs) and sensitivities (*S*) with respect to H_2_, confirm the stronger tendency of NO_2_ molecules to interact with the SnO_2_-QD/CuO surface as respect to less electronegative H_2_, as it will be discussed in the next paragraph.

Retrieving from literature data, Table 1 compares normalized relative responses – to 1 ppm NO_2_ and 100 ppm H_2_ (when available) of recently published CuO-based composites for gas sensing. Apart from Pd–CuO/rGO interfaces operating at 25 °C, our SnO_2_-QD/CuO yields comparable performances, eventually obtained at lower operating temperature as respect to the others.

**Table 1:** Comparison of the NO_2_ and H_2_ gas sensing response of CuO based composites normalized at 1 ppm NO_2_ and 100 ppm H_2_ (where applicable). Relative Response (RRs) indicated as: *R*_g_/*R*_a_ if *R*_g_ > *R*_a_; *R*_a_/*R*_g_ if *R*_g_ < *R*_a_

Sensing material	Gas concentration	Relative response RR [—]	OT [°C]	Ref.
**NO**_**2**_ **gas**				
SnO_*x*_/CuO	1 ppm	2.5	250	49
CuO/SnO_*x*_	1 ppm	1.5	250	49
CuO/ZnO	1 ppm	1.05	250	20
CuO/SnO_2_ nanoflowers	1 ppm	10	100	29
Pd–CuO/rGO	1 ppm	4.5	25	52
SnO_2_-QDs/CuO	1 ppm	2.5	100	This work

**H**_**2**_ **gas**				
CuO/Fe_2_O_4_	500 ppm	1.8	400	53
Nb_2_O_5_/CuO	0.5%	2	300	54
NiO/CuO	20 ppm	8	150	55
In_2_O_3_/CuO	400 ppm	1.3	350	56
TiO_2_/CuO/Cu_2_O	100 ppm	6	350	57
SnO_2_-QDs/CuO	100 ppm	1.7	100	This work

**Fig. 7:**
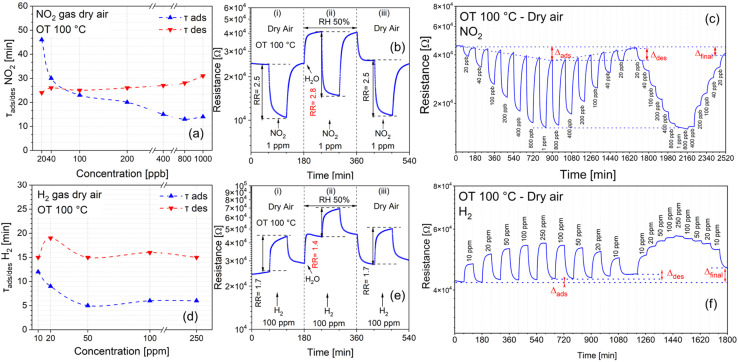
(a) and (d) Adsorption and desorption times of the SnO_2_-QD/CuO in dry air at an OT of 100 °C to NO_2_ (20 ppb to 1 ppm) and H_2_ (10 ppm to 250 ppm); (b) NO_2_ and (e) H_2_ cross-sensitivity to 50% relative humidity (RH). Each panel of figures (b)–(e) comprises: first panel (i), the response to 1 ppm NO_2_ (100 ppm H_2_) in dry air, second panel (ii), response 1 ppm NO_2_ (100 ppm H_2_) in 50% RH background, third panel (iii), response to 1 ppm NO_2_ (100 ppm H_2_), as to first panel, to check repeatability; (c) and (f) reproducibility and baseline recovery measured by exposing the SnO_2_-QDs/CuO to both pulse and cumulative NO_2_ (20 ppb to 1 ppm) and H_2_ (10–250 ppm) concentrations.

In addition, SnO_2_-QDs/CuO sensor's adsorption/desorption times (*τ*_ads_/*τ*_des_) to H_2_ are faster with respect to NO_2_. Comparing *τ*_ads_/*τ*_des_ to NO_2_ (Fig. 7a) and H_2_ (Fig. 7d), NO_2_ adsorption/desorption times are approximately two-fold those of H_2_, with desorption times always bigger than adsorption for both gases. Cross sensitivity tests were carried out to investigate the effect of humidity as interfering gas to the NO_2_ and H_2_ response, as shown in Fig. 7b and e. The standardized cross-sensitivity procedure comprises three steps. In a first step (panel i) SnO_2_-QD/CuO is exposed to 1 ppm NO_2_ and 100 ppm H_2_ in dry air; in a second step (panel ii) dry air background is replaced by 50% Relative Humidity (RH) humid-air while exposing the sensor to 1 ppm NO_2_ and 100 ppm H_2_, finally a third step (panel iii) is carried out in the same conditions of (panel i) to check for short term reproducibility. According to Fig. 7b and e, BLR of the SnO_2_-QD/CuO rapidly increases as soon as 50% RH is introduced, in line with p-type sensors response to humidity at operating at temperature higher than 25 °C.^58–60^ Significantly, relative response to 1 ppm NO_2_ increases from 2.5 ± 0.1 in dry air, to 2.8 ± 0.1 in 50% RH. That to 100 ppm H_2_ decreases from 1.7 ± 0.1 to 1.4 ± 0.1 respectively. This behavior is consistent with previous findings about a possible synergistic/antisynergistic effect of water vapor in the presence of oxidizing/reducing gases.^23,51^ Selectivity response (SR) shown in SI Fig. S3, highlights the superior ability of SnO_2_-QDs/CuO sensor to detect NO_2_ and H_2_ compared to 100 ppm NH_3_, 1% ethanol and 1% acetone. Notably, SR differs from humidity cross response (Fig. 7b and e), since it compares the sensor's ability to preferentially respond to a specific gas (*i.e.* 1 ppm NO_2_ target gas) while ignoring other gases or organic vapours under similar experimental conditions.

Finally, the short term reproducibility of the electrical response to NO_2_ and H_2_ to both pulsed (on/off) and cumulative (increasing/decreasing) concentration modes is shown in Fig. 7c and f. Specifically, under pulsed conditions, the baseline resistance (BLR) overall recovers its initial value after each desorption cycle in dry air, with a slight displacement from the BLR (as highlighted by *Δ*_ads_ for both gases), corresponding to higher gases' concentration. In a similar way, under cumulative stepwise adsorption/desorption modes, the NO_2_/H_2_ gases resistances taken at 1 ppm/100 ppm, perfectly matches the corresponding ones in pulse mode.

## 4. Gas sensing mechanism

The SnO_2_-QDs/CuO exhibits improved sensing performances with respect to its single counterparts, showing a prevailing a p-type response. Apart from the formation of p–n junctions, indeed SnO_2_ quantum dots significantly enhance the reacting surface area, increasing the number of available adsorption sites.

**Fig. 8:**
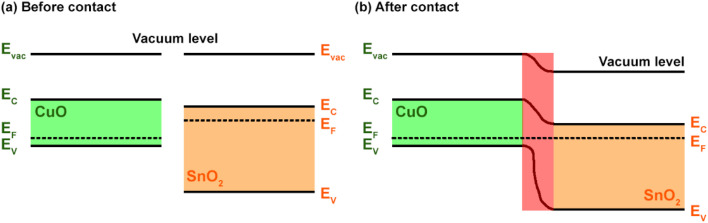
Schematization of the p–n heterojunction at the interface of CuO and SnO_2_ (a) before and (b) after contact.

The formation of p–n heterojunctions, as tentatively shown in Fig. 8, improves the gas sensing response of the SnO_2_-QD/CuO.^26,49,50^ Taking into account that CuO and SnO_2_ QDs yield band gaps (BG) of ≈1.8 eV and ≈4.1 eV (Fig. 5b), and assuming that CuO valence (VB) and conduction (CB) bands potentials are higher than those of SnO_2_,^49,50^ when the two materials are brought into contact, Fermi levels align and a typical *Z*-scheme heterojunction is build up.^50^ Specifically, electrons from SnO_2_ diffuse into CuO, and holes from CuO migrate into SnO_2_, forming a depletion region at the interface. Such charge redistribution leads to the establishment of an internal electric field, directed from SnO_2_ to CuO. As a result, a built-in potential barrier forms, causing an accumulation of electrons in the SnO_2_ conduction band and holes in the CuO valence band. Consequently, a majority of charge carriers (electrons for SnO_2_ and holes for CuO) are available to participate in the gas–surface reactions, yielding a stronger modulation of the electrical resistance in the presence of a target gas.

It turns out that the larger extension of the p-type CuO surface as respect to that of n-type SnO_2_-QDs, explains the overall p-type response of the SnO_2_/CuO. Given that, we may also estimate that the CuO surface of the heterojunction is the primarily reacting surface to NO_2_, H_2_ and H_2_O adsorbing molecules. Under these conditions at operating temperatures below 150 °C, the adsorption of oxygen on a doubly positively-charged oxygen vacancy 
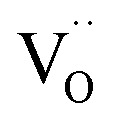
 leads to an increase of the holes h˙ concentration in p-type CuO (R1), resulting in a resistance decrease.R1
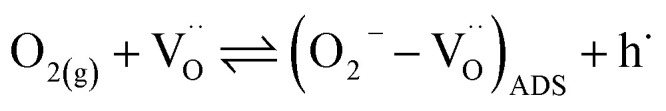


NO_2_ molecules, on the other hand, interacts with CuO by direct adsorption on free vacancy sites (R2).^23^ Being NO_2_ a strong electron-acceptor, electrons are released from the surface, leading to an increase of the h˙ concentration in the material, eventually decreasing the resistance (Fig. 6d).R2
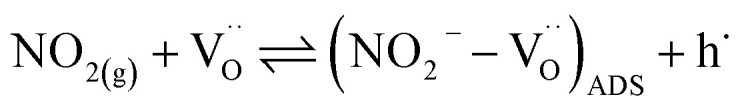


When a reducing gas like H_2_ is introduced, it generally reacts with adsorbed oxygen,^53^ decreasing h˙ concentration (R3), leading to a resistance increase (Fig. 6e).R3
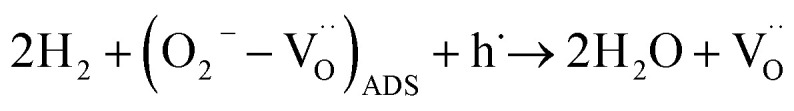


Regarding water interaction with CuO, a mechanism involving pre-adsorbed oxygen and Cu lattice atoms has been proposed,^58,61^ which yields a depletion of h˙ concentration (R4) and an increase of the sensor's resistance (Fig. 7b – panel (ii)).R4
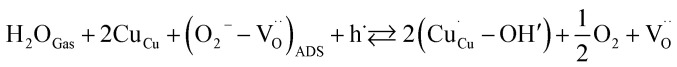


These mechanisms also provide a coherent explanation for the synergistic/antisynergistic effects induced by humidity in the presence of oxidizing or reducing gases. Specifically in Fig. 7b and e – panel (ii), we demonstrated that sensor's relative responses (RRs) to NO_2_/H_2_ gases in presence of 50% RH increases/decreases as respect to dry air background (see Fig. 7b and e – panel (i)), following a synergistic/antisynergistic response. In case of NO_2_, the synergistic response in 50% RH can be explained considering that as soon as 50% RH interacts with the sensor's surface (Fig. 7b – panel (ii)), reaction (R4) increases the concentration of 
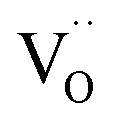
, boosting NO_2_ adsorption according to reaction (R2). Conversely, the antisynergistic response to H_2_ in the presence of 50% RH, can be described taking into account that as soon as 50% RH interacts with the sensor's surface (Fig. 7e – panel (ii)), reaction (R4) decreases the concentration of 
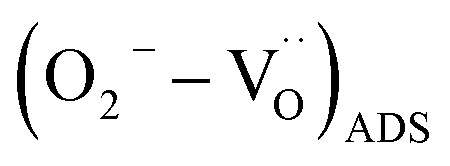
, therefore hampering H_2_ reduction according to reaction (R3). DFT atomistic simulations of NO_2_, H_2_ gases and H_2_O molecules adsorption over CuO and SnO_2_ metal oxides, highlight a substantial agreement with the ionosorption mechanism discussed in this paragraph. Specifically, it was confirmed the stronger oxidizing attitude of NO_2_ molecules to form NO_2_^−^ with oxygen-vacancy sites 
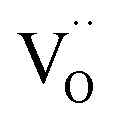
.^62,63^ Furthermore, while H_2_ adsorption on SnO_2_ is characterized by physisorption,^64^ its interaction with CuO follows a dissociative adsorption pathway.^65^ Accordingly, the weak interaction of H_2_ molecules predicted by DFT calculations, is congruent with the smaller sensor's signal variation to H_2_ compared to NO_2_, as illustrated in Fig. 6i. Finally, H_2_O vapor is reported to chemisorb on SnO_2_, forming two hydroxyl groups.^51^ On CuO, H_2_O adsorbs either chemically at Cu sites or dissociatively at oxygen-deficient regions.^66^ Both DFT models of H_2_O adsorption predict a decrease/increase of the electrical resistance for n-type SnO_2_ or p-type CuO, in agreement with experimental observations.

## 5. Conclusions

In this work, we have successfully demonstrated a simple and scalable synthesis of a novel heterojunction based on needle-like CuO decorated with SnO_2_ quantum dots (QDs, *d* = 3.9 nm) for NO_2_ and H_2_ gas sensing applications.

The composite exhibits markedly enhanced gas sensing performance compared to the individual components, with higher sensitivity toward the investigated species and excellent stability and reproducibility of the response. Notably, sensor operates efficiently at 100 °C OT, a significantly lower temperature compared to conventional metal oxide sensors, addressing a key limitation of to date gas sensing technologies. Ultimately, we studied a possible sensing mechanism explaining the role of the heterojunctions and possible gas–surface interaction, tentatively describing also the synergistic/antisynergistic effect played by relative humidity acting as interfering gas over the NO_2_/H_2_ responses.

In conclusion, these results point out the improving effect of the CuO and SnO_2_ QDs coupling, associated to efficient charge transfer and modulation the electronic structure of the sensing interface. Overall, this study provides a promising pathway toward the development of high-performance, low-temperature gas sensors based on engineered oxide.

## Conflicts of interest

The authors declare that they have no known competing financial interests or personal relationships that could have appeared to influence the work reported in this paper.

## Supplementary Material

RA-015-D5RA05533D-s001

## Data Availability

The data that support the findings of this study are available from the corresponding author upon reasonable request. Supplementary information is available. See DOI: https://doi.org/10.1039/d5ra05533d.
