# Supplementary material for: SnO2 quantum dot decoration of CuO nanoparticles with enhanced NO2 and H2 gas sensing response via p–n heterojunction interfaces
Source: RSC Adv. 2025 Oct 15;15(46):38750–61. doi: 10.1039/d5ra05533d (PMC12524114; doi:10.1039/d5ra05533d)
Supplement: RA-015-D5RA05533D-s001 [file RA-015-D5RA05533D-s001.pdf]

Supplementary Information (SI) for RSC Advances.
This journal is © The Royal Society of Chemistry 2025

SUPPORTING INFORMATION

SnO_2 quantum dots decoration of CuO nanoparticles with enhanced NO_2 and H_2 gas sensing response via *p-n* heterojunction interfaces

Valentina Paolucci[1], Thirugnanam Natarajan[1], Vittorio Ricci[1], Fabiola Ferrante[1], Carlo Cantalini[1]

[1]*Department of Industrial and Information Engineering and Economics, University of L'Aquila, I-67100 L'Aquila, Italy*

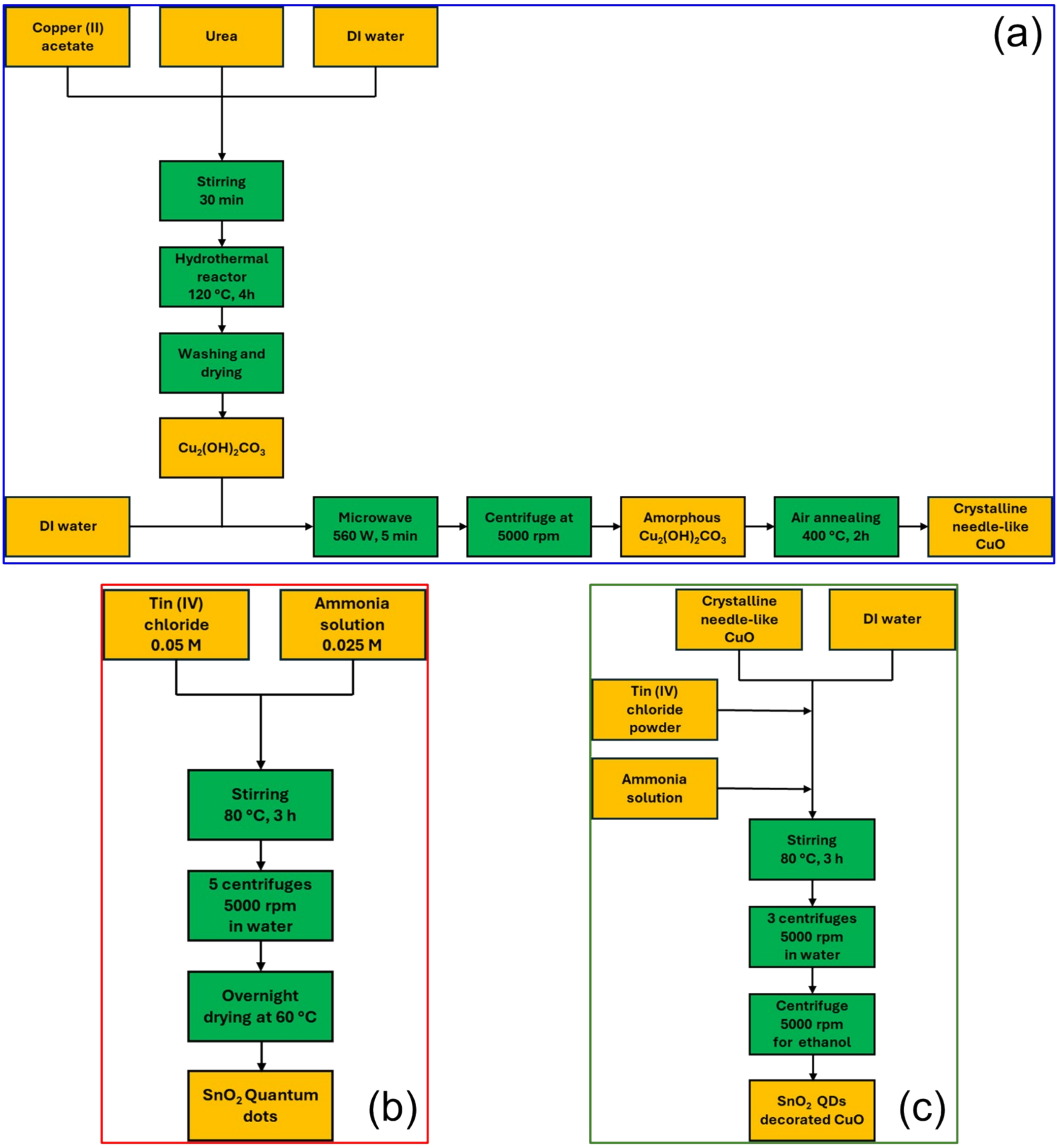

Figure S1. Flowcharts of the synthesis processes of: (a) Needle-like CuO nanoparticles; (b) SnO_2 quantum dots; (c) SnO_2 quantum dots-decorated CuO.

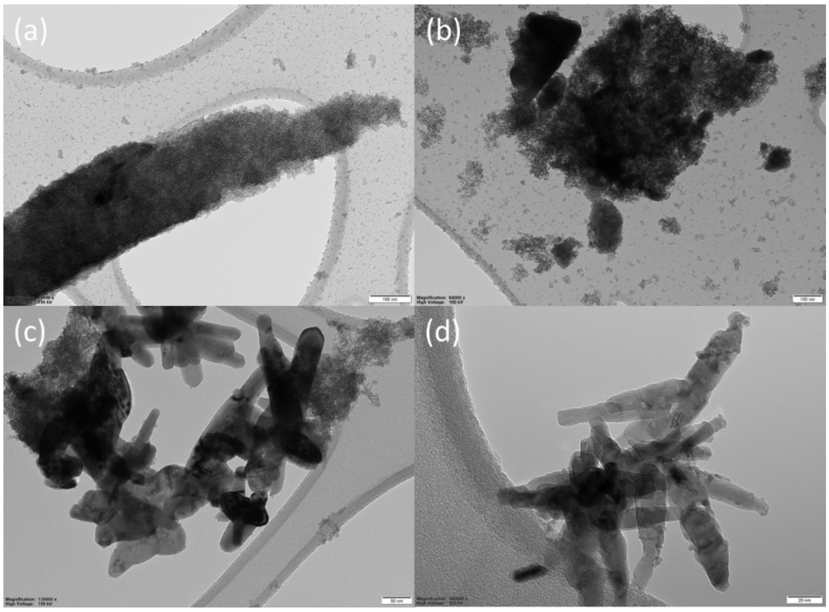

Figure S2. Optimization of the synthesis procedure. (a,b) Equimolar CuO and SnCl4: SnO2 QDs formed agglomerated clusters homogeneously dispersed in solution, with no preferential anchoring to CuO. (c) Reduced Sn precursor concentration: partial improvement in the flakes/dots ratio, although nucleation is still independent of CuO. (d) Optimized route: addition of SnCl4 directly to the CuO dispersion, followed by ammonia, yielding selective nucleation on CuO and the desired decoration with SnO2 dots.

The synthesis was first attempted by combining equimolar amounts of CuO and $SnCl_4$. In this case, $SnCl_4$ was initially dissolved in the 0.05 M ammonia solution and then mixed with the CuO dispersion (**Figure S1a-b**). The resulting SnO_2 quantum dots were agglomerated and these clusters homogeneously dispersed in substrate, rather than anchored to CuO, showing that nucleation occurred independently of the CuO surface. In addition, the amount of $SnCl_4$ used was found to be excessive. A second trial (**Figure S1c**), performed by reducing the Sn precursor concentration (1:10 as respect to CuO), led to a partial improvement in the flakes/dots ratio, although nucleation was still not correlated with CuO. The final optimization step was achieved by modifying the order of reagent addition: $SnCl_4$ (2.6 mg) was introduced directly into the CuO dispersion (25 mg in 20 ml DI water), and ammonia was added only afterwards. This simple adjustment successfully directed nucleation onto the CuO surface, leading to controlled decoration rather than uncontrolled nucleation in solution, therefore yielding the desired SnO_2 decoration (**Figure S1d**).

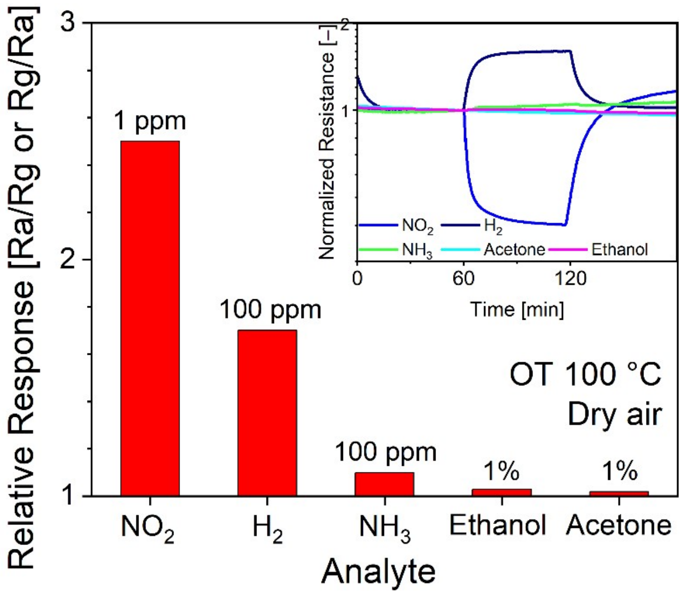

Figure S3. Selectivity responses to different concentrations of various gases and vapours (NO_2, H_2, NH_3, Ethanol and Acetone) measured at 100°C OT and dry air atmosphere.
